# Bactericidal nanotopography of titanium dental implants: in vitro and in vivo studies

**DOI:** 10.1007/s00784-025-06424-z

**Published:** 2025-06-25

**Authors:** Javier Gil, Mariano Sanz

**Affiliations:** 1 Bioinspired Oral Biomaterials and Interfaces, Department Ciencia e Ingeniería de Materiales, Escola d’Enginyeria Barcelona Est, Technical University of Catalonia, Barcelona, Spain; 2 ETEP (Etiology and Therapy of Periodontal and Peri-implant Diseases) Research Group, Faculty of Dentistry, Complutense University, Madrid, Spain

**Keywords:** Peri-implantitis, Passivation, Titanium, Bactericidal effect, Dental implant, Fatigue

## Abstract

**Objective:**

A new passivation method for titanium dental implants has been studied, where the nanotextured layer features spikes that provide a high bactericidal capacity without compromising the degree of osseointegration of the dental implants.

**Materials and methods:**

This layer has been obtained through a sulfuric acid treatment with hydrogen peroxide. It has been characterized using electron microscopy, the roughness was determined by confocal microscopy and wettability and surface energy assessed through contact angle. The incorporation of hydrogen was assessed using a fusion spectrometer. Fatigue behavior was evaluated with a servo-hydraulic testing machine. The adhesion of human osteoblastic cells SaOs-2 at 3 and 7 days was measured, and the level of mineralization was analyzed by alkaline phosphatase levels. Bacterial colonization assays were conducted using four strains to assess their bactericidal capacity. Implants were inserted into rabbit tibiae. After 21 days, the animals were sacrificed, and bone index contact determined.

**Results:**

A uniform surface created by nanospikes was obtained, exhibiting the same roughness as the control implant, no hydrogen was incorporated inside the titanium. The fatigue behavior showed no variation compared to the control. An increased wettability and higher surface energy compared to the control implant were noted. Enhanced osteoblastic adhesion was observed for the nanospikes surface in comparison with control at 3 days, with a significant level of alkaline phosphatase at 14 days, indicating a good degree of mineralization. The bactericidal capacity of nanospike surface is evidenced showing reductions ranging from 70 to 90%. In vivo tests demonstrate higher bone contact index values for dental implants with nanospikes (56%) compared to the control (41%).

**Conclusions:**

The surface formed by nanospikes maintains the mechanical properties of the control and improves the wettability of the surface which improves the behavior of the osteoblasts generating a better osseointegration. At the same time, it has a high bactericidal capacity that prevents microbiological colonization.

**Clinical relevance:**

Peri-implantitis has become one of the major problems for the success of implant dentistry and this new surface may be a solution for the prevention of the disease.

## Introduction

Titanium dental implants are very successful in the functional rehabilitation of the oral cavity. This fact is due to the good mechanical properties, corrosion resistance, biocompatibility and especially the high osseointegration capacity, which facilitates a strong implant-bone tissue integration [1, 2].

 However, peri-implantitis continues to be a significant factor contributing to dental implant failure. Globally, this condition is projected to result in an estimated expenditure of $2.3 billion in 2024 related to subsequent treatment [3, 4]. The prevalence of peri-implantitis among dental implants ranges from 12 to 24%, equating to over 375,000 affected implants annually [5].

Peri-implantitis has emerged as a public health concern in dentistry. It is well recognized that peri-implantitis is an inflammatory lesion of the mucosa surrounding the implant with progressive loss of the peri-implant supporting bone. Bacterial colonization may lead to microorganisms’ infiltration of the peri-implant space and form biofilms on the titanium surface [6–8]. Peri-implant pathogens contribute to the destruction of peri-implant tissues. These microorganisms in addition to generating an inflammatory response can proliferate and generate the formation of biofilms. These facts generate the progression and severity of periodontal disease since biofilms are resistant to antibiotics and regular dental cleaning [9, 10].

The technique of implantoplasty, which involves the mechanization of the surface of the dental implant, results in mechanical weakness, a loss of corrosion resistance, and, more concerning, the release of particles of various sizes that are not aspirated during treatment and remain in the biological tissues. Various studies have demonstrated the cytotoxic effects of these particles, particularly those of nanometric size [11–13]. Additionally, research by Kotsakis et al. has indicated that titanium machined by implantoplasty leads to inflammation, creating an anaerobic environment around the titanium that reduces titanium oxide to pure titanium [14]. As the oxygen content increases, titanium oxidizes but does not reach the stoichiometry of TiO_2_, forming mixed oxides that have been shown to be toxic [14, 15].

This leads us to conclude that clinicians in implantology and scientists in materials science and engineering must collaborate to develop a passivation layer on titanium with stable bacteriostatic and/or bactericidal properties throughout the lifespan of the dental implant. This is essential to inhibit biofilm formation and, subsequently, prevent peri-implantitis.

In this research it is about obtaining a titanium dioxide passivation layer that has an acicular nanotopography that causes the penetration of bacteria causing their death and avoiding or at least strongly preventing the formation of biofilm on the surface of the titanium. This surface modification from a smooth passivation layer to one formed by nanopillars is obtained by chemical reaction based on an attack with sulfuric acid and hydrogen peroxide and has an indefinite bactericidal effect. New surface does not affect long-term mechanical performance. The physicochemical characteristics of the surface have been studied and the nanotopography causes an increase in wettability and polar component of the surface energy that will cause an increase in osteoblastic activity. This effect on these cells has been seen on cell adhesion and mineralization. As a consequence, we have been able to verify the increase of bone index contact on the nanospike surfaces with respect to the control. Likewise, we were able to verify very important reductions of four bacterial strains generating periodontal disease in values of approximately 70%. We believe that this surface may be promising for reducing peri-implantitis.

## Materials and methods

### Material specimens

Dental implants made of cp. titanium grade 3 produced by Klockner (Escaldes Engordany, Andorra) were utilized. The implants feature a rough surface obtained through grit-blasting, using the projection of abrasive particles (Al_2_O_3_) sized between 200 and 400 μm at a pressure of 2.5 bars. An acid etching treatment created two types of surfaces:

- Standard implants, as received, serve as the negative control.
- Test implants using the new passivation method (Spikes) based on immersion in sulfuric acid and hydrogen peroxide for 2 h.

### Roughness

White light interferometry (Wyko NT1100 Optical Interferometer, Veeco Instruments, USA), operating in vertical scanning interferometry mode, was employed to produce, evaluate, and quantify the topography. The vertical resolution (≈ 2 nm). The analysis area measured 124.4 × 94.6 μm. Data analysis was conducted using Wyko Vision 32 (Veeco Instruments, USA), which facilitates the application of a Gaussian filter to differentiate waviness and form from roughness. Five distinct specimens of each surface were measured to determine the amplitude parameter (Sa), the spacing parameter (Sm), and the hybrid parameter (Index area).

### Wettability and surface energy

Contact angle analysis was performed on 6 samples (3 for the control samples and 3 for the nanospike surface) using ultrapure distilled water (Millipore Milli-Q, Merck Millipore Corporation, Darmstadt, Germany) and formamide (Contact Angle System OCA15plus-Dataphysics, Filderstadt, Germany), with the corresponding data analyzed using SCA20 (Dataphysics, Filderstadt, Germany). Measurements of the contact angle were performed via the sessile drop method. Drops were generated using a micrometric syringe and deposited on discs. A total of 3 µL of distilled water and 1 µL of formamide were applied to each sample at a rate of 200 µL/min. Finally, the surface energy was calculated by applying the Owens, Wendt, Rabel, and Kaelble (OWRK) equation to the wettability values obtained from distilled water and formamide [16–19].

1 $$\:{\gamma\:}_{L}\left(1+\text{cos}\theta\:\right)=2\left({\left({\gamma\:}_{L}^{d}{\gamma\:}_{S}^{d}\right)}^{\raisebox{1ex}{$1$}\!\left/\:\!\raisebox{-1ex}{$2$}\right.}+{\left({\gamma\:}_{L}^{p}{\gamma\:}_{S}^{p}\right)}^{\raisebox{1ex}{$1$}\!\left/\:\!\raisebox{-1ex}{$2$}\right.}\right)$$

where γ^d^ and γ^p^ represent the dispersive and polar components of the liquid surface tension (γ_L_). θ indicates the contact angle between the liquid (L) and the solid (S).

Data were analyzed using SCA 20 software (Dataphysics). Three measurements were taken for three different samples in each series.

### Hydrogen content

The hydrogen concentration within the control and nanotextured titanium was analyzed because interstitial hydrogen could infiltrate the structure. This diffusion of hydrogen into the interior can lead to the hydrogen atoms reacting with each other at the boundaries of the crystalline grains, forming the H_2_ molecule and increasing in volume which causes cracks between the crystals due to insufficient accommodation of the hydrogen volume changes. This analysis was conducted using an Inert Gas Fusion LECO TCH600 (Standard QA-PRO-7452-037-INTA) to determine the hydrogen content in the titanium alloy’s interior. IGF-MS was performed on five specimens of each surface [20].

### Fatigue

The fatigue behavior and limit of the prototype were established using Wöhler curves (stress-number of cycles), which illustrate the relationship between the amplitude of cyclical stresses and the number of cycles until failure. During testing, the implant-abutment system was exposed to both cyclical compressive and lateral forces without any lateral constraints. Five hundred specimens were tested.

The tests were conducted using the MTS Bionix 858 servo-hydraulic testing, which was specially designed and equipped with a 25 kN PC runningTESTAR II ^®^ software and according to the guidelines previously published by the FDA in the Class II Special Controls Guidance Documents: Dental Implants Abutments and the ISO 14801:2007.

**Fig. 1:**
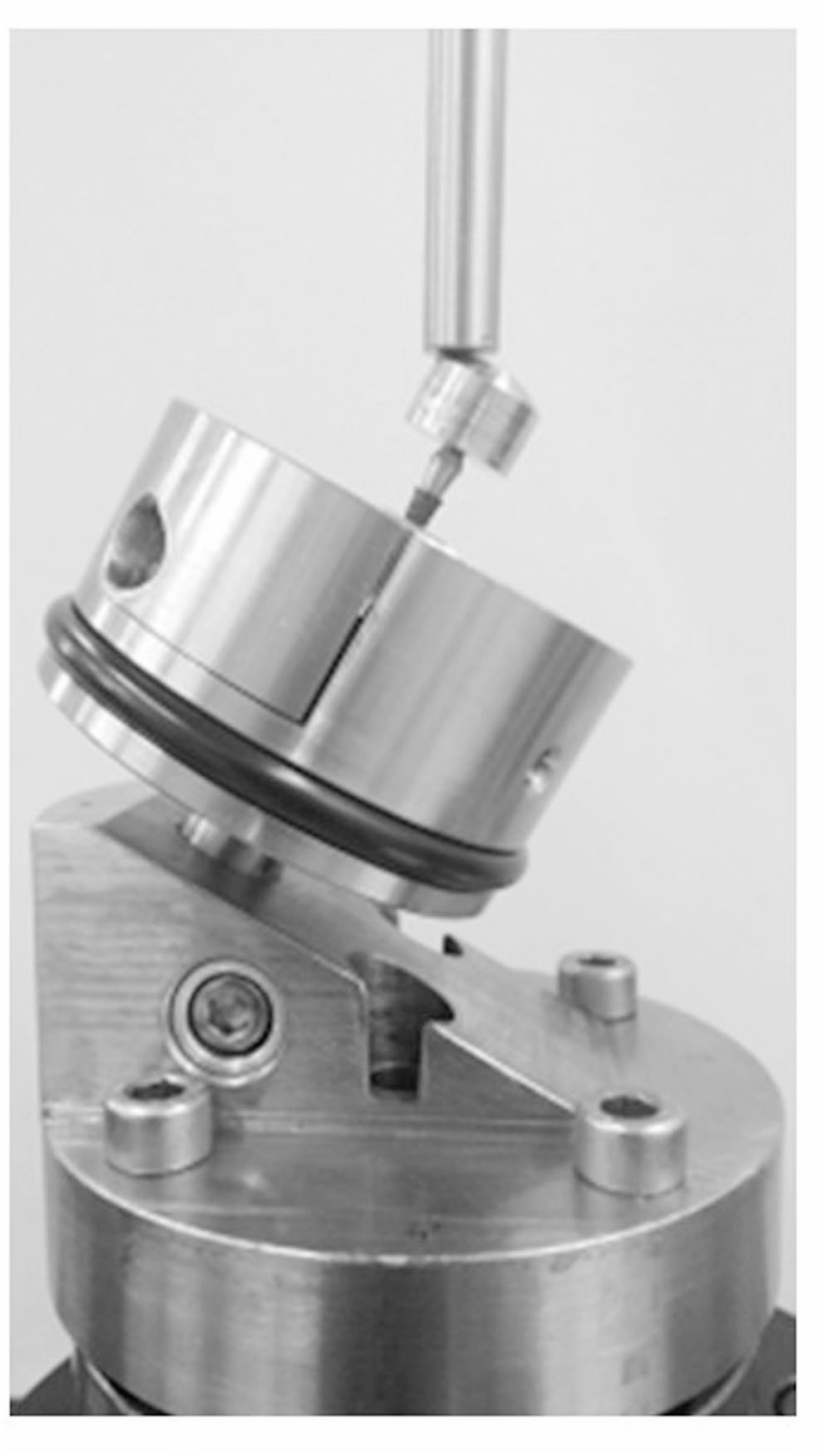
Fatigue set-up with tri-axes (tensile-compression and torsion) to simulate the oral mechanical behavior

The tested implants supported an abutment that was in line with the axis of the implant. The testing setup clamped the implant so that the implant’s long axis made a 30° angle with the loading direction of the testing machine and, consequently a flexural load was applied. The implants were fixed with a 30-degree inclination from the z-axis of the traction-compression machine. A 30-degree angle to the z-axis of the tensile-compression machine is recommended by FDA standards as the most unfavorable position. Additionally, the implant was placed 3 mm below the expected crestal bone level, simulating 3 mm of bone resorption. Figure 1 shows the experimental setup.

First, five resistance tests were conducted at the selected inclination to determine the yield strength of the material and the ultimate flexural strength. The various percentages of yield strength obtained from these results, ranging from 60 to 90%, were subsequently used to perform fatigue tests to measure the number of cycles until fracture.

The goal is to determine the level of stress at which the sample endures five million cycles, which will be regarded as the fatigue limit. Seven of the tests conducted to ascertain the level of stress analyzed the fatigue limit, while three tests evaluated the other tested stresses. The implants were subjected to a sinusoidal fatigue function at a frequency of 15 Hz, and the relationship between maximum and minimum applied stress was 10%. The tests took place at room temperature. The data obtained were represented as the number of cycles to failure as a function of applied stress.

### Scanning electron microscopy

A high-resolution Field Emission Scanning Electron Microscope (FE-SEM) (FEI Nova Nano SEM 230 Hillsboro, OR, US) was utilized to examine the micro and nanostructure of the surface.

### Cell viability and differentiation

For the in vitro studies, osteoblastic cells (SaOS-2; ATCC, Manassas, VA, USA) were utilized. They were cultured in McCoy’s modified 5 A medium, which was supplemented with 10% fetal bovine serum (FBS, Gibco, New York, NY, USA), 1% penicillin/streptomycin, 2 mM (Invitrogen, Carlsbad, CA, USA), and 1% sodium pyruvate (Invitrogen, Carlsbad, CA, USA). Cultures were grown at 37 °C in a 5% CO_2_ incubator under humidified conditions. The samples were analysed at 3 and 7 days. The total number of samples was 40 (10 samples x 2 surfaces x 2 times).

Confluent cells were incubated with TrypLE (Invitrogen, Carlsbad, CA, USA) for 1 min to detach them from the flask. Subsequently, 5,000 cells were seeded onto each disc and incubated at 37 °C. After 3 and 21 days of incubation, the samples were washed with PBS and transferred to a new plate to conduct the metabolic activity assay using Alamar Blue (Invitrogen-Thermo Fisher Scientific, Waltham, MA, USA), following the protocol. Briefly, the reagent was prepared and pipetted to cover the samples, and the percentage of Alamar Blue reduction was determined after 4 h of incubation at 37 °C, using the Alamar Blue solution as a blank.

To study osteogenic differentiation, alkaline phosphatase (ALP) activity was determined for 20 samples (10 for each surface) using the Sensolyte pNPP alkaline phosphatase colorimetric assay (Anaspec, Fremont, CA, USA). ALP was measured at a wavelength of 495 nm, and detection was performed with a conventional ELx800 microplate reader (Bio-Tek Instruments, Inc. Winooski, VT, USA).

### Bacterial adhesion

Four types of bacteria, including *Streptococcus gordonii* (CECT 804), *Streptococcus oralis* (CECT 907), *Actinomyces viscosus* (CECT 488), and *Enterococcus faecalis* (CECT 795), were utilized for the bacterial adhesion test. Tryptic soy broth (TSB) served as the culture medium for *Streptococcus gordonii*, while brain heart infusion (BHI) was employed for the others. We tested 10 samples per group for each bacterial strains and for each surface (10 samples x 4 bacteria strains x 2 surfaces = 80 samples).

The culture media and materials were sterilized by autoclaving at 121 °C for 30 min. The samples were sterilized by washing them with ethanol for 5 min, followed by three washes with water for 5 min each, and exposure to ultraviolet light for 15 min on each side.

The bacteria inocula were prepared by suspending the bacteria in 5 mL of the appropriate media and allowing them to grow overnight at 37 °C. The medium was then diluted to 600 nm to achieve an optical density of 0.2. The sterile samples were placed in 24-well plates, covered with 700 µL of the diluted bacterial suspension, and incubated at 37 °C for 2 h to assess bacterial adhesion. As a positive control, 700 µL of bacterial suspension was added to an empty well plate. After this incubation period, the samples were washed with PBS and transferred to a new 24-well plate for metabolic and live–dead assays.

For the metabolic assay, three samples and the positive controls were incubated with 650 µL of resazurin sodium salt at a concentration of 25 µg/mL in PBS (Sigma-Aldrich, St. Louis, MO, USA) at 37 °C until the positive control became saturated. Subsequently, 100 µL was used to measure the absorbance at 570 and 600 nm, which was then used to calculate the reduction percentage.

In the case of the Live–Dead assay, the remaining two samples were stained with the LIVE/DEAD^®^ BackLight™ Bacterial Viability Kit solution (Thermo Fisher Scientific, Waltham, MA, USA). Briefly, the two reagents were diluted at a ratio of 1.5 µL of reagent per mL of PBS. The samples were then covered with 650 µL of the solution and incubated for 15 min at 37 °C. Afterward, the samples were washed twice with PBS, and images were acquired from three different regions using a confocal laser microscope at 64× (Leica Dmi8, Wetzlar, Germany), employing excitation/emission wavelengths of 589/615 nm for dead cells and 495/520 nm for live cells.

### In vivo tests

#### Animals

Ten healthy adult New Zealand white rabbits (Universitat Autònoma de Barcelona, Cerdanyola, Spain), aged 6 to 7 months and with an average weight of 5 kg, were utilized after receiving approval from the University’s ethical committee (08/14/LU-002). The procedures took place at the Animal Experimentation Facility of the Universitat Autònoma de Barcelona, Spain. All experiments adhered to the Spanish Government Guide and the European Guide for Animal Care. The rabbits were housed in enriched cages, which allowed for normal activity, and were monitored daily by trained staff to assess any changes in their general health. The animals had unrestricted access to food and tap water.

#### Implants

This study used two different surfaces of the identical dental implants: 20 implants with control surface and 20 with nanospikes topography. Their diameter was 4.1 mm, and the length was 10 mm.

#### Surgical procedures

All surgical procedures were conducted in an operating room under sterile conditions and with general anesthesia induced and maintained at a concentration of 2.5–4% isoflurane (Isoba-vet, Schering-Plough, Madrid, Spain). The animals were initially sedated with a combination of medetomidine (50 mg/kg/i.m., Domtor, Esteve, Barcelona, Spain) and ketamine (25 mg/kg/i.m., Imalgène 1000, Merial, Toulouse, France). Throughout the anesthesia, the animals were continuously monitored by a veterinarian categorized as B or C.

The animal received antibiotic prophylaxis for one week with enrofloxacin (15 mg/kg, subcutaneously, once daily, Ganadexil 5%, Invesa, Barcelona, Spain) and pain was controlled with meloxicam (0.2 mg/kg, subcutaneously, for three days, Metacam, Boehringer Ingelheim, Barcelona, Spain).

Each animal received two implants (control and nanospike) in the right and left femur, totaling 40 implants across 10 animals. The experimental site was located on both distal lateral condyles of the femur. After 21 days of quarantine, the animals were placed under general anesthesia. Following shaving and disinfection, the femoral condyles were exposed via a lateral longitudinal incision. Implant bed preparations were carried out according to the manufacturers’ recommendations. Finally, the muscle, subcutaneous tissue, and skin were sutured in layers with absorbable sutures (Vicryl 4 − 0, Ethicon, New Jersey, USA).

21 days later, rabbits were painlessly sacrificed by a sodium pentobarbital overdose (100 mg/Kg/i.v., Dolethal, Vétoquinol, Madrid, Spain) after sedation with ketamine and medetomidine.

### Histological and histomorphometric analyses

Blocks containing the implant and the distal, distracted femur bone, along with hard and soft tissues surrounding the implant, were obtained using an oscillating saw, then fixed and identified. These blocks were dehydrated in a series of graded ethanol (70–100%) and infiltrated with different mixtures of ethanol and glycolmethacrylate (Technovit 7200 VLC, Heraus Kulzer, Werheim, Germany), following guidelines previously published [21]. The samples were then polymerized and heated at 37ºC for 24 h to ensure complete polymerization.

Longitudinal sections (central sections) in the direction of 200 microns were created from the implants using a band saw and mechanically micropolished (Exakt Apparatebau, Norderstedt, Germany) with 1200 and 4000 grit silicon carbide papers (Struers, Copenhagen, Denmark) until samples with a thickness of approximately 40 μm were obtained. The slides were stained using the Levai-Laczkó method [15, 21, 22] for both histological and histomorphometric analysis.

Quantitative and semiquantitative histology was performed using a motorized light microscope and a digital camera connected to a PC-based image capture system (BX51, DP71, Olympus Corporation, Japan). The regions of interest were peri-implant tissues. Image analysis was conducted based on the color and shape of these structures, differentiating new and lamellar bone from connective and vascular tissues (Adobe Photoshop, CA, USA). Parameters were evaluated and measured by a masked examiner using the PC-based image analysis program CellSens 1.5 (Olympus Corporation, Japan). The implant surface in contact with mineralized bone, known as “bone to implant contact” (BIC), was calculated as a percentage.

### Statistical analysis

The data was statistically analyzed using Student’s t-tests, one-way ANOVA tables, and Tukey’s multiple comparison tests to evaluate any statistically significant differences between the samples at alpha = 0.05.

Table 1 summarizes the number of samples/dental implants for each of the experiments performed and for each of the surfaces (control and nanospikes). Electron microscopy observations were performed on the samples on which we determined the roughness since it is a non-destructive test.

**Table 1:**
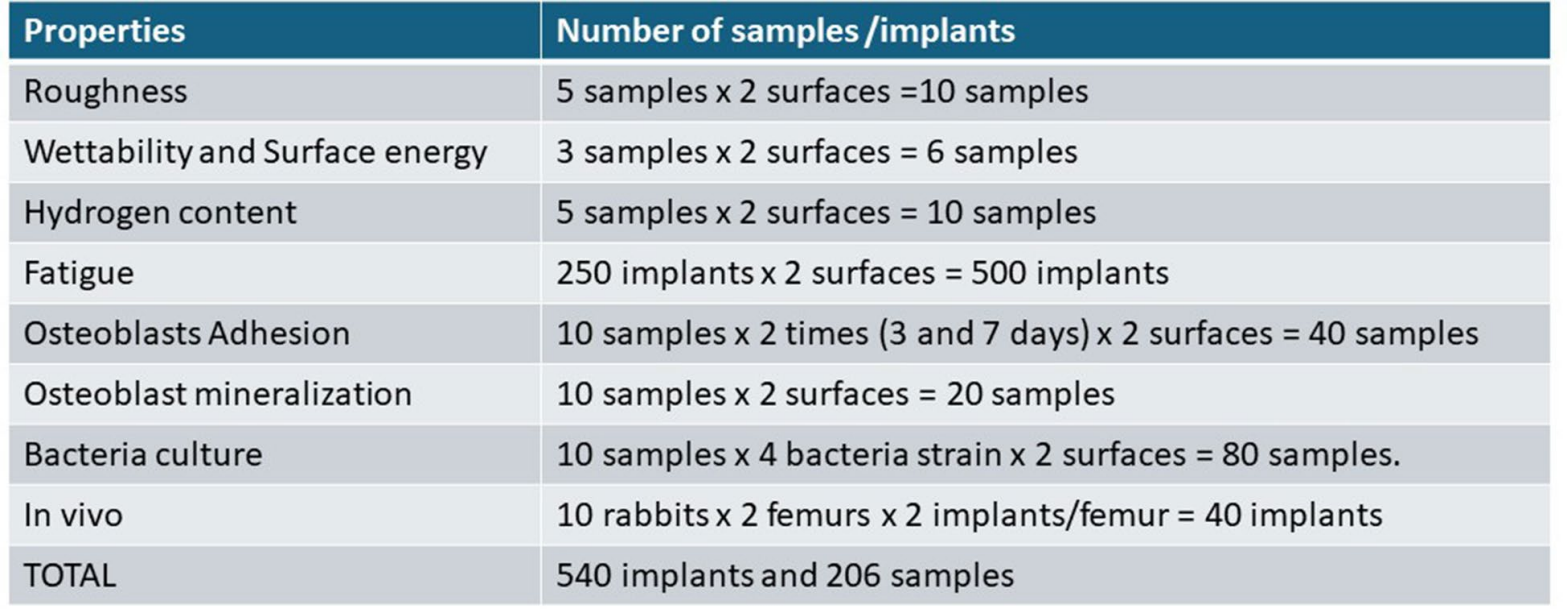
Number of samples and dental implants used for the characterization

## Results

**Fig. 2:**
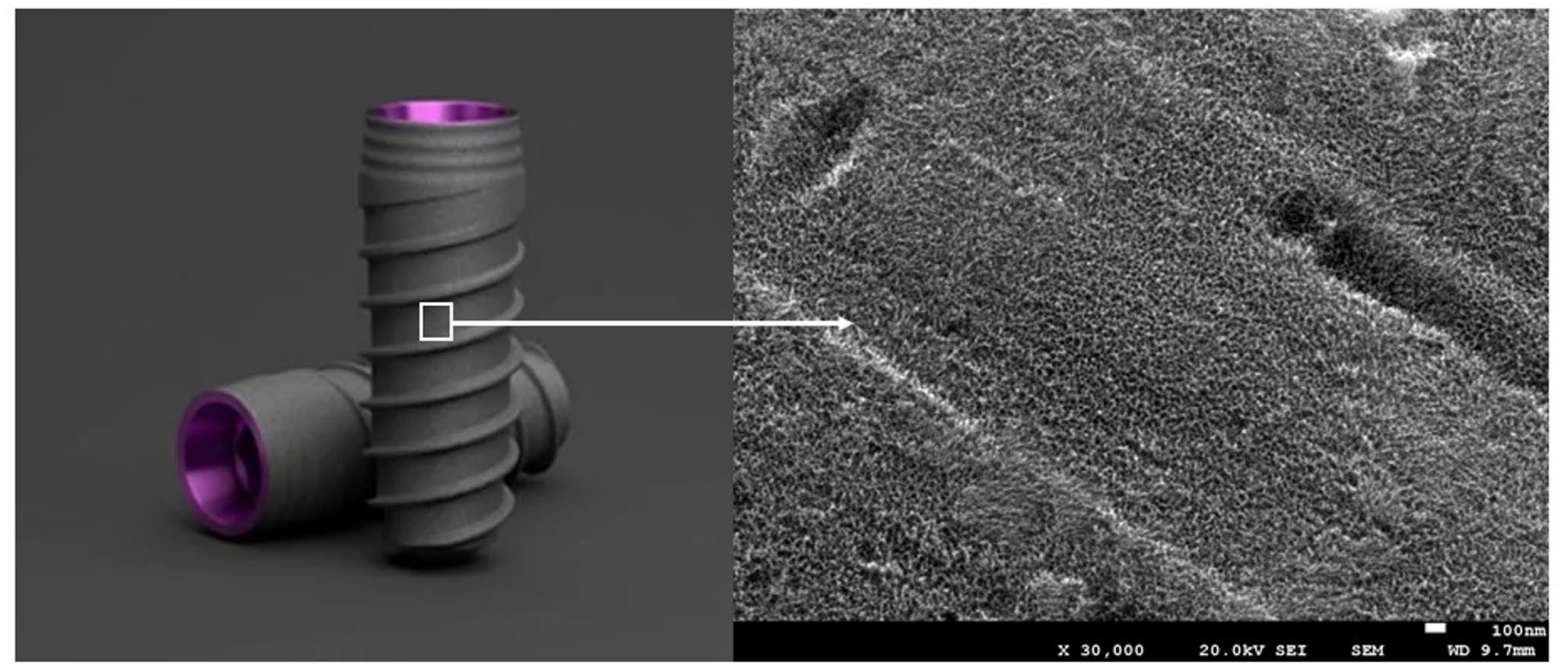
Nanospikes topography on the surface of the dental implants

Figure 2 illustrates the nano-topography resulting from treatment with sulfuric acid and hydrogen peroxide, highlighting the texture of the nanopillars.

This microstructure does not lead to a variation in surface roughness, as shown in Table 2; the nanotexture mirrors the surface topography since it is not a coating but rather a passivation reaction involving a liquid mixture of acids that attacks all parts uniformly.

**Table 2:** Mean values ± standard deviation of the roughness parameters for all titanium surfaces studied. Sa: average roughness, sm: quadratic mean of the roughness, index area: surface area at the lateral resolution of the measured surface as compared to that of a perfectly smooth surface. The results do not present statistically differences (*P* < 0.05)

Surface	Sa (µm) ± SD	Sm (µm) ± SD	Index Area ± SD
Control	1.91 ± 0.12	2.34 ± 0.02	1.59 ± 0.01
Nanospikes	2.01 ± 0.15	2.67 ± 1.00	1.56 ± 0.05

**Fig. 3:**
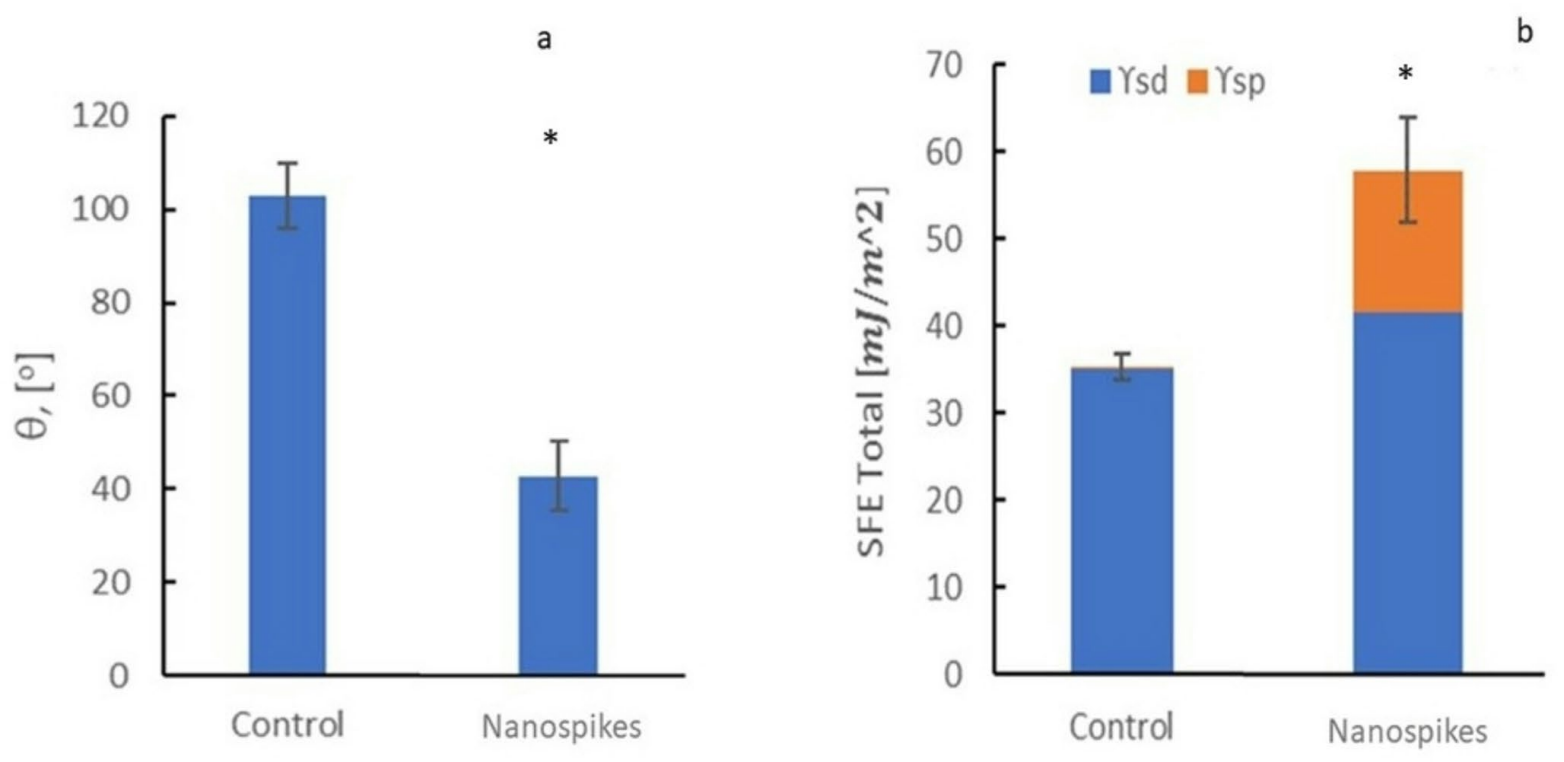
**a**. Contact angles for each surface. **b**. Total surface energy with dispersive (γ_sd_) and polar (γ_sp_) components. Asterisk indicates significant difference (*p* < 0.05)

In Fig. 3a, we observe the wettability values for the control sample, and the one composed of nanopillars, noting that the latter is 20º more hydrophilic than the control, making it more hydrophilic. Additionally, Fig. 3b shows that the titanium treated with acid has a higher surface energy and contributes more to the total surface energy of the polar component.

The results of hydrogen incorporation showed no statistically significant differences with *p* < 0.05 with compared the control and nanospikes samples: The control samples showed values of 34.0 ± 15.0 ppm and the nanospikes showed 32.0 ± 17.0 ppm.

**Fig. 4:**
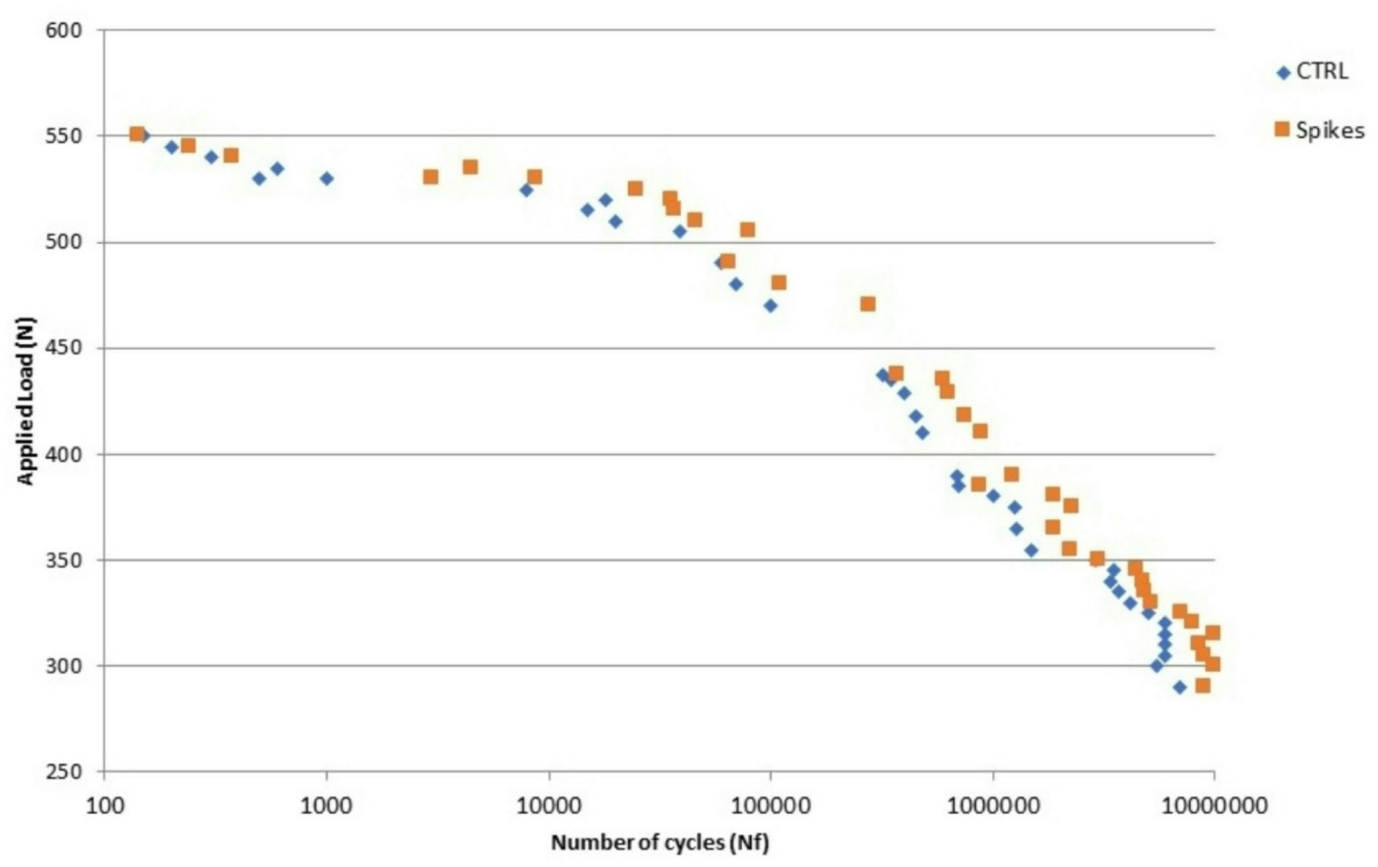
Applied load versus number of cycles to fracture for control and nanospikes dental implants

The fatigue tests are shown in Fig. 4, which displays the number of cycles to fracture at various levels of mechanical load. It is evident that the control curves and those of the nanospike samples are nearly identical. Additionally, at 350 N, the asymptote is reached, indicating that the life of the implant is infinite, thereby ensuring the strong mechanical response of the treated dental implants.

**Fig. 5:**
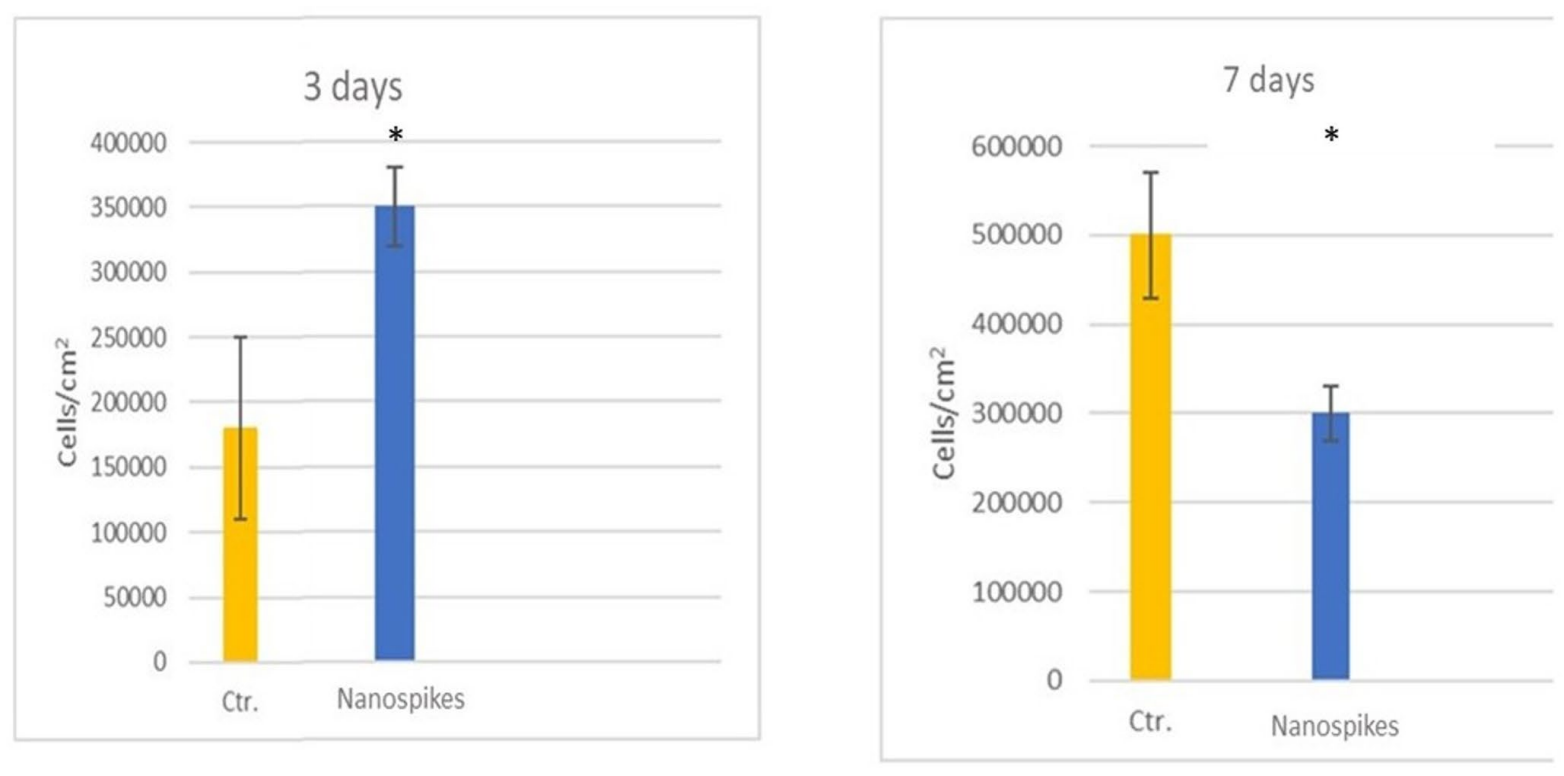
Osteoblastic activity at 3 and 7 days. Asterisk indicates significant difference (*p* < 0.05)

**Fig. 6:**
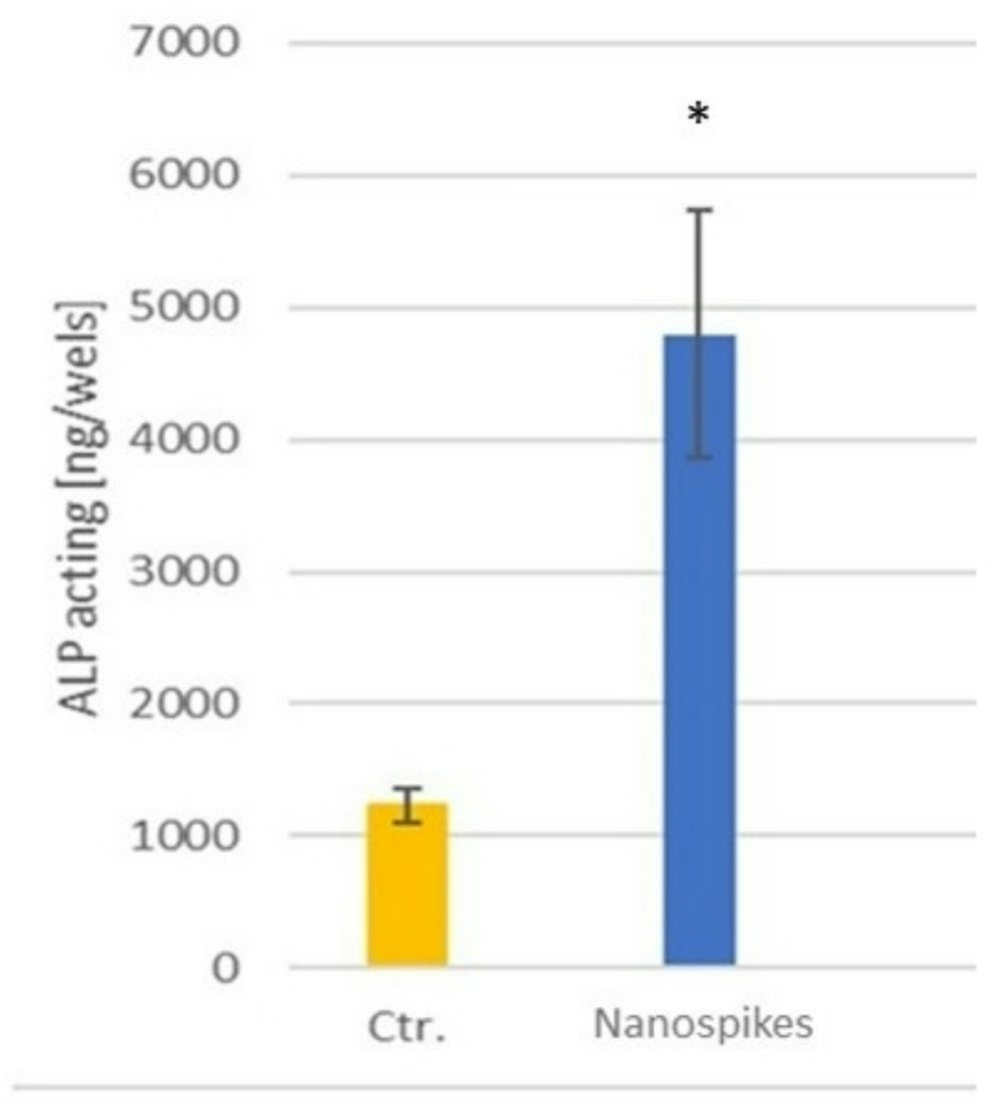
Alkaline phosphatase at 14 days. Asterisk indicates significant difference (*p* < 0.05)

The osteoblastic activity can be observed in Fig. 5, which shows the adhesion of the osteoblasts at 3 and 7 days. At 3 days, the adhesion to the nanospike surfaces is statistically significantly higher than that of the control implant, with *p* < 0.05. However, at 7 days, there is a decrease in the number of adherent osteoblasts, suggesting that in the case of the sample formed by nanospikes, the cells are in the process of proliferation or mineralization. This is supported by the alkaline phosphatase levels measured at 14 days, where the sample with nanospikes exhibits statistically significant differences (*p* < 0.05) compared to the control samples, as can be seen in Fig. 6.

**Fig. 7:**
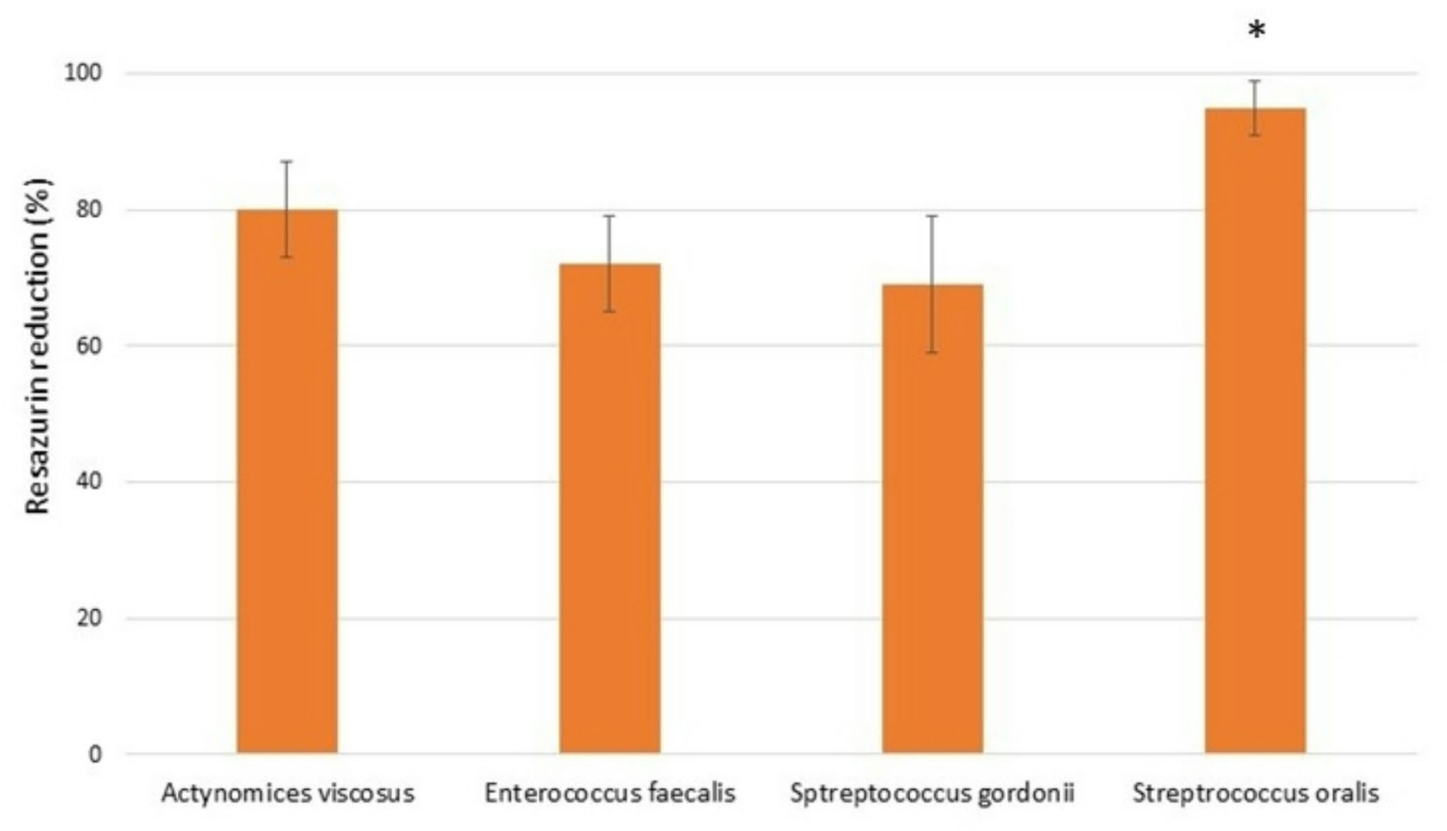
Resazurin reduction in percentage for the 4 bacteria strains studied for the nanospike surface in relatio to the control. Asterisk indicates significant difference (*p* < 0.05)

The microbiological behavior shows the percentage of difference between nanospike and control for all the strains studied, as illustrated in the results of Fig. 7. Colony reductions are observed at 95% for *Streptococcus oralis*, 80% for *Actinomyces viscosus*, 72% for *Enterococcus faecalis*, and 69% for *Streptococcus gordonii*.

As can be seen from the results, the effect of the nanospikes surface is more effective for *Streptococcus oralis* bacteria than the others studied. However, the results are very encouraging since in all cases there is a reduction of more than 69% in comparison to control.

**Fig. 8:**
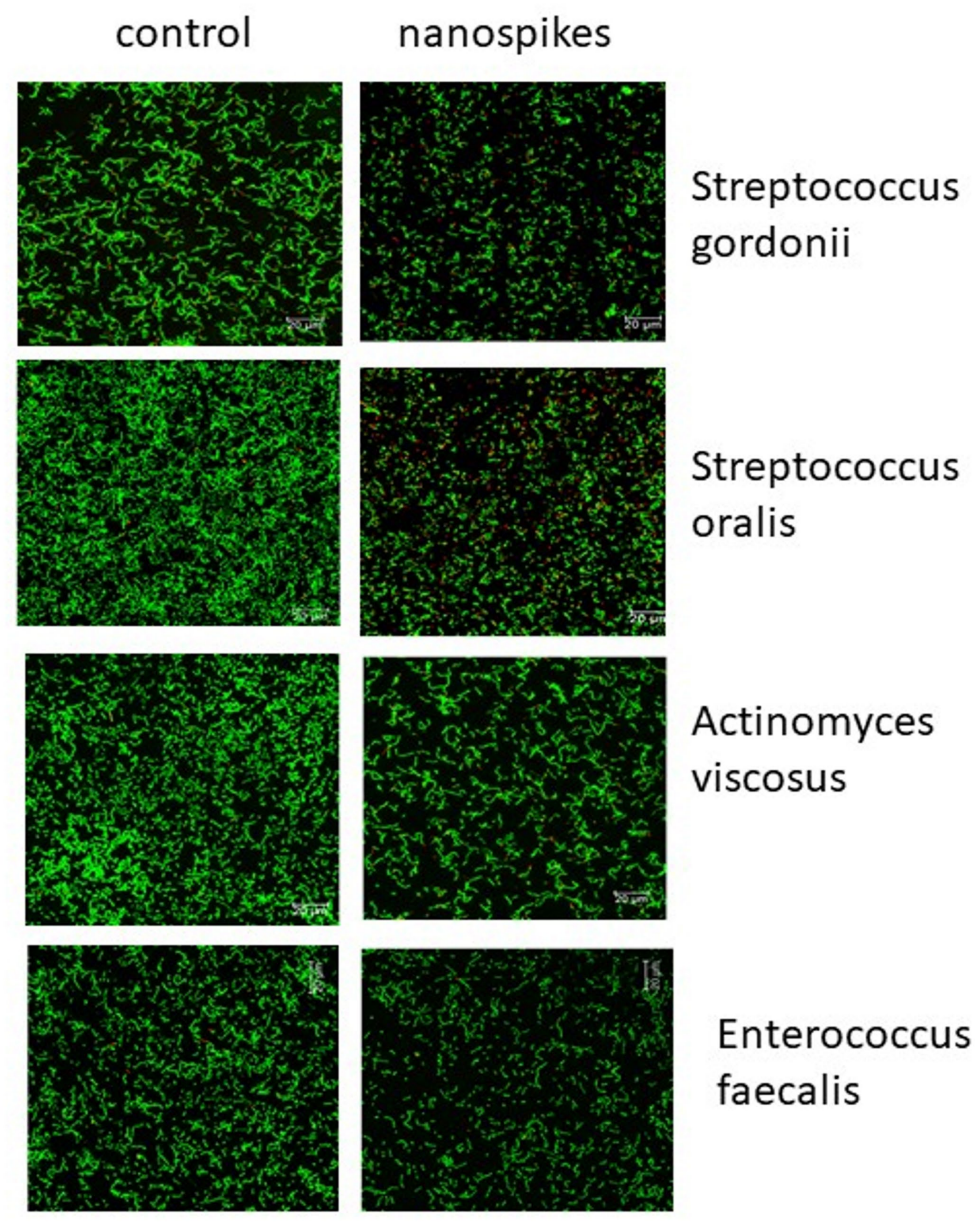
Live and dead of the different bacteria strains studied. Colour green are alive and red are dead

Figure 8 displays the LIVE/DEAD assays, which reflect the results of the metabolic activity.

**Fig. 9:**
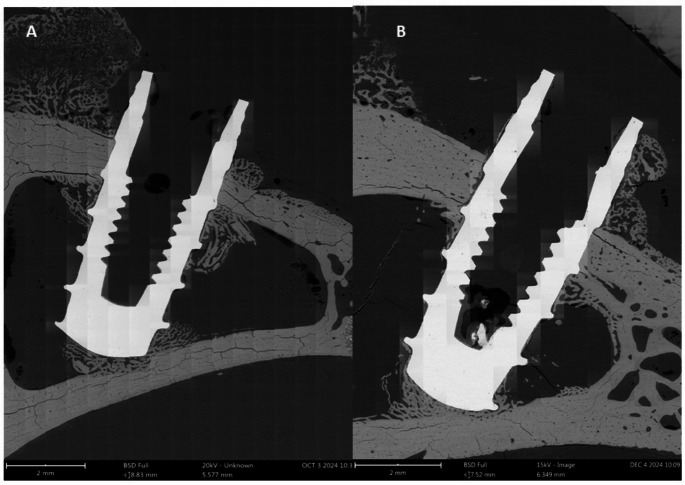
Macroimages obtained by scanning electron microscope for different surfaces. **A**. Control surface **B**. Nanospikes surface after 21 days implanted

All dental implants placed in the rabbits succeeded, and the animals’ behavior and inflammation levels remained normal. After 21 days, they were euthanized, and the osseointegration study was conducted using the Bone index contact parameter, which yielded values of 54 ± 8% for the surfaces with nanospikes and 41 ± 9% for the control dental implants. The differences were statistically significant, with *p* < 0.05. In Fig. 9 can be observed the different osseointegration between control and nanospikes surfaces.

## Discussion

It is known that roughness plays a significant role in osseointegration capacity; numerous studies indicate that topography influences adhesion, proliferation, and osteoblastic differentiation behavior [22–24]. Research has determined that optimal values for cell behavior fall between 2 and 4 micrometers Ra. However, these same values also favor bacterial colonization. After extensive studies, a suitable compromise value for dental implants has been established at Ra between 1.4 and 2 micrometers [23, 25]. It is well known that bacteria do not easily colonize smooth surfaces. Nevertheless, it has been shown that very low Ra roughness of less than 0.5 micrometers exhibit less bacterial colonization than smooth surfaces [26]. Implementing these roughness levels on titanium surfaces is technologically challenging due to the lack of small-sized abrasive particles suitable for projection onto titanium. Although nanostructures can be created on titanium surfaces using lasers, this approach is not economically favorable. Furthermore, laser application can cause fusions in the titanium that lead to a sudden decline in mechanical properties. This fine roughness is inspired by cicada wings [27], which prevent attacks from other insects and serve a defensive purpose.

In acid attacks designed to form a passivation layer on titanium, concentrated sulfuric acid, along with an oxidizing effect, is known to create surface topographies in conjunction with hydrogen peroxide. Other studies have successfully established the optimal conditions for producing a nanotexture characterized by the formation of nanospikes that could serve as a bactericidal agent for dental implants and their prostheses [28, 29]. This passivation layer increases corrosion resistance, cleans the surface, and decreases the release of titanium ions into the physiological environment, resulting in a lasting bactericidal effect [30–32]. The remarkable aspect of this nanotexture is that it is not merely a coating but rather a new morphology of titanium oxide that forms spontaneously on the surface, thus maintaining the material’s high biocompatibility. This treatment of nanospikes is long-range since there is no drug release but the bactericidal effect is due to its topography. The spikes penetrate the bacterial membrane causing the death of the microorganism [33, 34].

This new topography can also be generated on titanium and titanium alloy abutments (Ti6Al4V) and will have the same effect as on the dental implant. Therefore, bacteria will be killed by adsorbing on the surface and will not be able to generate colonies leading to biofilm formation. One of the limitations of the nanospike surface is that it cannot select bacteria and there are bacteria that aid the digestive processes that are present in the mouth that are susceptible to die. There will also be no cell death, neither fibroblasts nor osteoblasts due to the large size of the cells relative to the bacteria and that is why the nanopillars will not affect the integrity of the cells.

In this study, we have been able to verify how the nanotexture decreases the contact angle, making the surface more hydrophilic and thus enhancing wettability [35, 36]. This results in easier protein adsorption on the titanium surface, which, due to its strong polar contribution to surface energy, favors the adsorption of proteins such as fibronectin, a precursor to the adhesion of osteoblastic cells [37]. This relationship is evidenced by the rapid osteoblastic adhesion observed at three days and the significant mineralization achieved at 14 days, indicating the favorable condition of the osteoblasts and the ease of bone tissue formation. These in vitro results are further supported by histological studies of the implants inserted into the tibiae of rabbits, which show an increase in the bone index contact of more than 10 points in samples with nanospikes compared to the control implants. The histology indicates good bone quality, and at no point was there any infection in the implants, demonstrating a normal degree of inflammation for this intervention.

The titanium nanopillars damaged the bacteria while remaining compatible with osteoblastic cells, which appeared intact after 24 h of attachment [35]. The destruction of bacteria depends on the characteristics of different species of microorganisms, including size, shape, membrane configuration, composition, and cell rigidity, ultimately determining the response to mechanical disruption [36]. The contact between bacteria and surface nanofeatures may lead to cell death [37, 38], secondary to bacterial membrane stretching or puncture caused by the contact between the bacterium and the features, especially when the latter display high aspect ratios and sharp shapes. Velic et al. [39] found that the membrane rupturing mechanism is more related to envelope strain and predominantly occurs at the tip of the pillars. Xue et al. [40] developed a mechanical model to explain the antibacterial effect shown by nanostructures such as the nanopillars found on cicada wings. According to this model, the geometric parameters of the surface features determine the bactericidal nature of the surface, and gravity and nonspecific forces, such as van der Waals, contribute to cell destruction by rupture, rendering Gram-negative bacteria more susceptible to this situation [41–43].

One of the limitations of this study is that bacterial colonization, in general, does not begin in the dental implant but in the oral cavity producing a bacterial filtration that generates the formation of biofilms also located on the surface of the dental implant. It would be advisable to carry out the studies on the prosthetic abutments, but this bactericidal treatment can also be obtained on these surfaces since in general the abutments are made of titanium or Ti6Al4V where the attack of sulfuric acid with hydrogen peroxide will produce the same surface of nanopillars. The results we have obtained in this research are fully valid for the pillars. Another limitation is that it would be advisable to place dental implants in animals with infection to determine the behavior of this new treatment. However, the ethics commission did not allow this research since it was obligatory to give antibiotics to the animal and this fact would not allow to determine the efficacy of the nanospikes treatment. It would also be necessary to carry out cultures with biofilms of bacteria associated with periodontal infections to determine the behavior of this treatment. This work is planned to be carried out in the near future with bioreactors simulating oral conditions with the formation of biofilms in the laboratories of the Faculty of Dentistry of the Complutense University in Madrid.

It would be very interesting to determine whether it is possible to obtain this type of surface on other metals used in prostheses such as CrCo or stainless steels, as well as on ceramics such as zirconia or calcium phosphate-based materials for bone regeneration. This topic of study is another aspect to be addressed in the near future.

## Conclusions

This new topography of the passivation layers on titanium dental implants, characterized by nanospikes, demonstrates a high bactericidal capacity against the four strains studied, with less CFU ranging from 70 to 90%. Additionally, the new nanotexture maintains the roughness of the dental implant, enhances wettability, and exhibits surface energy with a certain polar character that promotes osteoblastic adhesion and mineralization. Studies have shown that the mechanical fatigue properties remain unchanged by the acid treatment applied to the titanium, and there is no hydrogen incorporation within the titanium implant. In vivo tests corroborate the in vitro studies, showing higher bone contact index values compared to control dental implants. This innovative treatment may serve as a strong candidate for dental implants with enhanced bactericidal capacity and improved osseointegration.

## Data Availability

No datasets were generated or analysed during the current study.
